# Supplementary material for: Phosphorylation of Thr9 Affects the Folding Landscape of the N-Terminal Segment of Human AGT Enhancing Protein Aggregation of Disease-Causing Mutants
Source: Molecules. 2022 Dec 10;27(24):8762. doi: 10.3390/molecules27248762 (PMC9786777; doi:10.3390/molecules27248762)
Supplement: Supplementary file 1 [file molecules-27-08762-s001.zip › molecules-2065647-supplementary.pdf]

Supplementary Information

Figure S1. Conformational changes in the NTT-AGT peptides with 3.9 M TFE at different temperatures. Lines show thermal denaturation scans. Open circles show results from wavelength scans at 10 ºC steps from an independent experiment for each variant. Open squares show results from wavelength scans registered upon heating up to 85-95ºC and cooling down to 5 ºC (reversibility tests). The scan rate was 1.5 ºC·min^{-1}. The NTT-AGT WT peptide was prepared at 50 µM in K-phosphate 20 mM pH 7.4 with 0-7.6 M TFE.

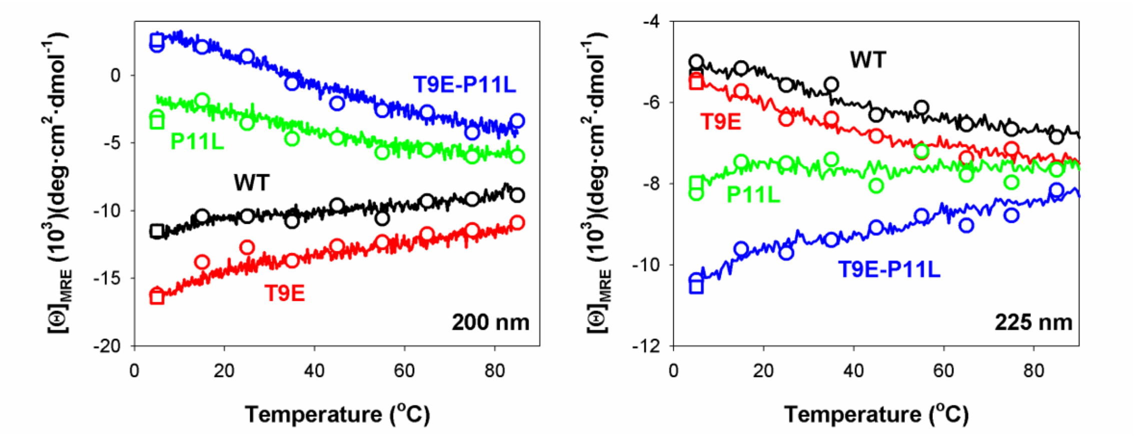

Figure S2. Titration of the WT peptide with TFE at 20 ºC. The WT peptide was prepared at 50 µM in K-phosphate 20 mM pH 7.4 with 0-7.6 M TFE.

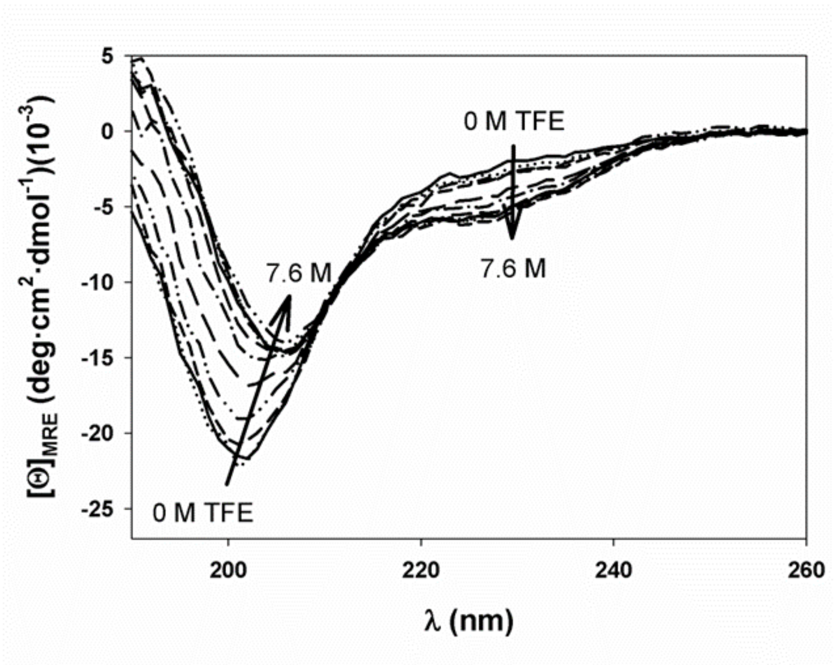

Figure S3. Selected regions in the ^{1}H-1D-NMR spectra of NTT-AGT peptides. A) Amide region; B) Methyl and alkyl regions.

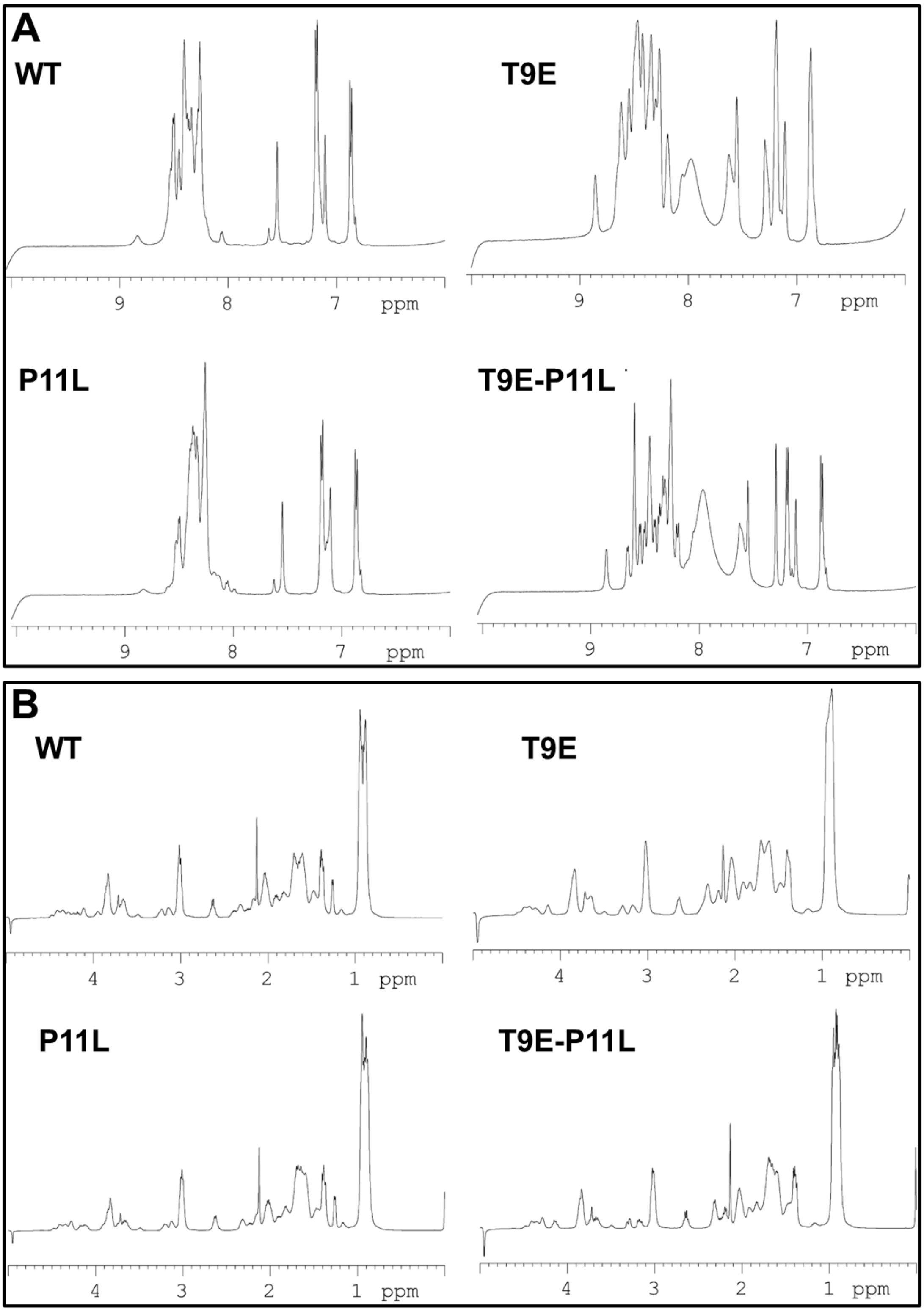

Figure S4. The amide regions of the^{1}H-1D-NMR spectra of NTT-AGT peptides in the presence of 30 % TFE. The spectrum of the T9E mutant was too broad under these conditions.

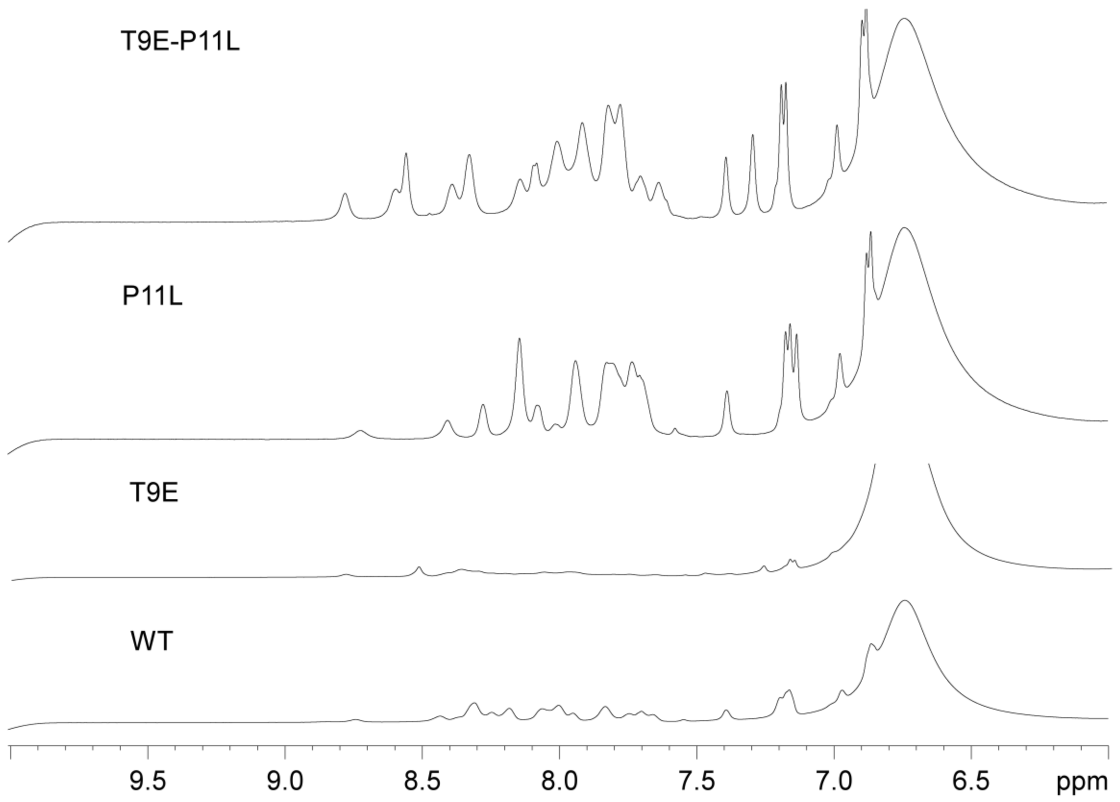

Figure S5. The amide region of the ^{1}H-2D-NOESY spectra of wild-type NTT-AGT peptide. The NOESY spectra comprising the NH (F2)-Hα (F1) for the wild type in water (black) and in the presence of the co-solvent (red)

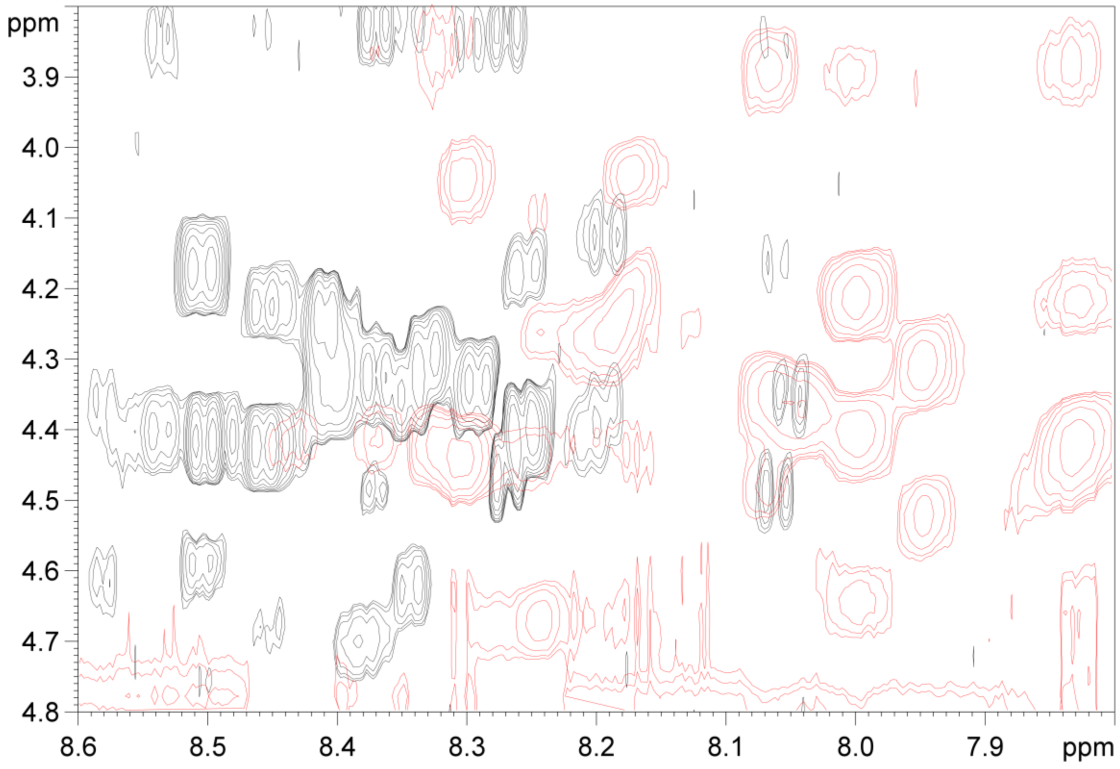

Figure S6. PLP binding kinetics to apo-AGT variants (WT, T9A and T9E). A) Pseudo first-order binding kinetics (protein and PLP concentrations were 0.5 µM and 80 µM, respectively). The lines are best-fits to a single exponential function that provide k_{obs}. B) Linear dependence of k_{obs} on [PLP] concentration. The slope of these fits provides the k_{on} and the intercept with the y-axis provides the k_{off}.

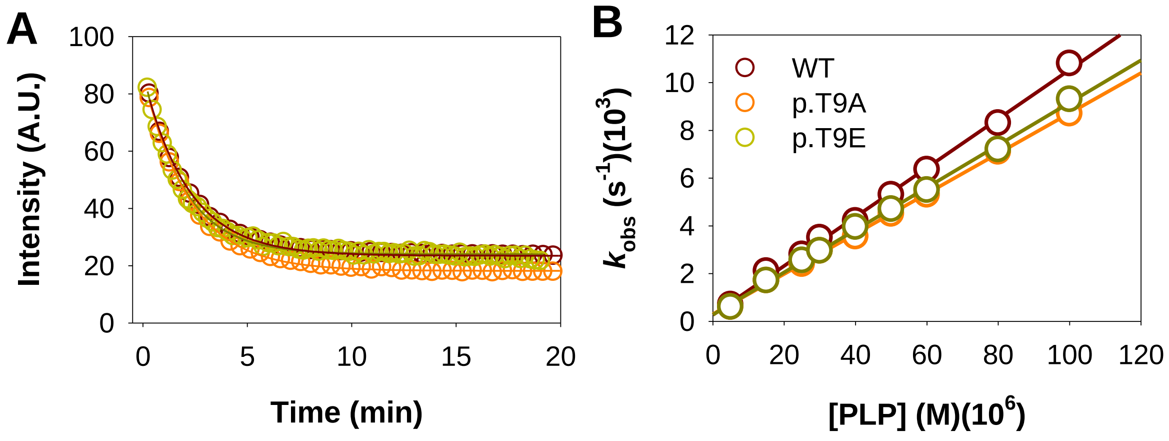

Table S1. Chemical shifts (δ, ppm from TSP) of NTT-AGT WT in aqueous solution (pH 7.2, 10 °C)

	NH	Hα	Hβ2	Hβ3	Hγ2	Hγ3	Hδ2	Hδ3	Hε	Hε	Hζ
Met1		4.10	2.18		2.63						
Ala2		4.42	1.39 (Me)								
Ser3	8.54	4.39	3.84								
His4	8.55 (broad)	4.70	3.14				7.16 (C4H); 8.27 (C2H) (broad)				
Lys5	8.34	4.63	1.77		1.48						
Leu6[a]	8.29	4.31	1.64		1.64		0.92 (Me)				
Leu7 [a]	8.29	4.31	1.64		1.64		0.92 (Me)				
Val8	8.25	4.18	2.06		0.90 (Me)						
Thr9	8.50	4.60	4.10		1.26 (Me)						
Pro10			2.05; 1.88		2.39		3.97; 3.71				
Pro11		4.42	2.04; 1.91		2.32		3.66				
Lys12	8.46	4.22	1.77		1.50						
Ala13	8.41	4.28	1.39 (Me)								
Leu14	8.33	4.36	1.61		1.61		0.92 (Me)				
Leu15[a]	8.41	4.34	1.61		1.61		0.91 (Me)				

Lys16	8.38	4.48	1.75	1.35			
Pro17		4.45	2.94; 1.85	2.24		3.87; 3.69	
Leu18	8.51	4.31	1.63	1.63		0.93 (Me)	
Ser19	8.37	4.47	3.82				
Ile20[b]	8.07	4.18	1.73; 082 (Me)	1.48	1.08	0.82 (Me)	
	8.27	4.18	1.84; 0.91 (Me)	1.48	1.13	0.91 (Me)	
Pro21		4.42	2.05	2.27		3.65	
Tyr22	8.45	4.54	3.09			7.18	6.86

[a]Tentatively assigned.
[b] Two set of signals were observed due to cis-trans isomerization of Pro21.

Table S2. Chemical shifts (δ, ppm from TSP) of NTT-AGT P11L in aqueous solution (pH 7.2, 10 °C)

	NH	Hα	Hβ2	Hβ3	Hγ2	Hγ3	Hδ2	Hδ3	Hε	Hε	Hζ
Met1		4.10	2.18		2.63						
Ala2	8.80	4.42	1.42 (Me)								
Ser3	8.54	4.42	3.86								
His4	8.51		3.17					7.10 (C4H); 8.26 (C2H)			
Lys5											
Leu6											
Leu7			1.62								
Val8	8.24	4.18	2.05		0.90 (Me)						
Thr9	8.43	4.61	4.15		1.26 (Me)						
Pro10		4.37	2.03; 1.91		2.32		3.94; 3.75				
Leu11	8.50	4.31	1.65		1.65		0.93 (Me)				
Lys12	8.35	4.28	1.75								
Ala13	8.36	4.28	1.42 (Me)								
Leu14											
Leu15											

Lys16	8.37	4.48	1.75					
Pro17		4.42	2.02; 1.89		2.30		3.83; 3.66	
Leu18	8.41	4.29	1.62		1.62		0.91 (Me)	
Ser19	8.38	4.51	3.85					
Ile20[a]	8.06	4.18	1.75; 0.84 (Me)	1.48		1.12	0.84 (Me)	
	8.24	4.28	1.88; 0.90 (Me)			1.19	0.90 (Me)	
Pro21		4.39	1.99; 1.84		2.24		3.88; 3.69	
Tyr22	8.24	4.50	3.03				7.18	6.86

[a] Two set of signals were observed due to cis-trans isomerization of Pro21.

Table S3. Chemical shifts (δ, ppm from TSP) of NTT-AGT T9E in aqueous solution (pH 7.2, 10 °C)

	**NH**	**Hα**	**Hβ2**	**Hβ3**	**Hγ2**	**Hγ3**	**Hδ2**	**Hδ3**	**Hε**	**Hε**	**Hζ**
Met1		4.13	2.16		2.63						
Ala2	8.85	4.42	1.42 (Me)								
Ser3	8.54	4.40	3.87								
His4[a]	8.51		3.30; 3.18				7.28 (C4H); 8.56 (C2H)				
Lys5	8.45	4.55	1.77								
Leu6											
Leu7		4.40	1.62								
Val8	8.18	4.14	2.04		0.89 (Me)						
Glu9	8.61	4.59	2.01; 1.87		2.34						
Pro10		4.37	2.04		2.37		3.90; 3.71				
Pro11											
Lys12			1.75								
Ala13	8.42	4.27	1.38 (Me)								
Leu14	8.33	4.34	1.67		1.45		0.93 (Me)				
Leu15											
Lys16											

Pro17		4.42	1.89	2.24		3.84; 3.68	
Leu18	8.48	4.32	1.61	1.61		0.91 (Me)	
Ser19	8.36	4.49	3.84				
Ile20[b]	8.04	4.16	1.74; 0.86 (Me)	1.49	1.07	0.84 (Me)	
	8.26	4.28	1.83; 0.90 (Me)	1.49	1.18	0.90 (Me)	
Pro21						3.83	
Tyr22	8.26	4.50	3.03			7.18	6.87

[a] The signal was very broad.

[b] Two set of signals were observed due to cis-trans isomerization of Pro21..

Table S4. Chemical shifts (δ, ppm from TSP) of NTT-AGT T9E-P11L in aqueous solution (pH 7.2, 10 °C)

	NH	Hα	Hβ2	Hβ3	Hγ2	Hγ3	Hδ2	Hδ3	Hε	Hε	Hζ
Met1		4.17	2.18		2.63						
Ala2	8.86	4.42	1.42 (Me)								
Ser3	8.54	4.41	3.82								
His4	8.66	4.73	3.30; 3.18				7.28 (C4H); 8.61 (C2H)				
Lys5	8.46	4.56	1.64								
Leu6											
Leu7			1.62								
Val8	8.20	4.12	2.03		0.91 (Me)						
Glu9	8.60	4.59	2.05; 1.92		2.33						
Pro10		4.37	1.89		2.30		3.82				
Leu11	8.50	4.31	1.67		1.67		0.92 (Me)				
Lys12	8.33	4.63	1.76								
Ala13	8.47	4.30	1.42 (Me)								
Leu14											
Leu15		4.31	1.61				0.93 (Me)				

Lys16	8.37	4.46	1.75						
Pro17		4.40	2.03; 1.90		2.30			3.82	
Leu18	8.45	4.40	1.62		1.62			0.92 (Me)	
Ser19[a]	8.38	4.51	3.82						
	8.31	4.49	3.86						
Ile20[a]	8.07	4.18	1.74; 0.85 (Me)	1.48		1.07		0.85 (Me)	
	8.27	4.28	1.83; 0.90 (Me)	1.64		1.15		0.90 (Me)	
Pro21		4.42	1.96		2.24		3.83		
Tyr22	8.26	4.48	3.04					7.18	6.86

[a] Two set of signals were observed due to cis-trans isomerization of Pro21..

Table S5. Chemical shifts (δ, ppm from TSP) of NTT-AGT WT in 30 % TFE (pH 7.2, 10 °C)

	NH	Hα	Hβ2	Hβ3	Hγ2	Hγ3	Hδ2	Hδ3	Hε	Hε	Hζ
Met1		4.16	2.18		2.63						
Ala2	8.75	4.42	1.45 (Me)								
Ser3	8.31	4.43	3.88								
His4	8.44	4.67	3.30, 3.22				7.21 (C4H); 8.33 (C2H)				
Lys5	8.24	4.26	1.77								
Leu6	8.00[a]	4.35	1.76		1.61		0.90 (Me)				
Leu7	8.20	4.27	1.64		1.64		0.93 (Me)				
Val8	7.83	4.20	2.11		0.93 (Me)						
Thr9	8.00	4.37	4.15		1.25 (Me)						
Pro10		4.43[a]	2.08		2.35		3.90; 3.65				
Pro11		4.46	2.10		2.38		3.86; 3.60				
Lys12	8.30	4.09	1.86		1.54						
Ala13	8.17	4.22	1.42 (Me)								
Leu14	7.74	4.34	1.71		1.60		0.94 (Me)				
Leu15	7.67	4.29	1.74		1.56		0.86 (Me)				

Lys16	7.94	4.52	1.83					
Pro17		4.42	2.09; 1.92		2.33		3.70; 3.67	
Leu18	8.00	4.34	1.75		1.66		0.94 (Me)	
Ser19	8.06	4.47	3.89					
Ile20	7.84	4.19	1.86; 094 (Me)	1.56		1.18	0.94 (Me)	
Pro21		4.37	2.16		2.32		3.90;3.64	
Tyr22	7.70	4.50	3.04				7.17	6.85

[a]Tentatively assigned.

Table S6. Chemical shifts (δ, ppm from TSP) of NTT-AGT P11L in 30 % TFE (pH 7.2, 10 °C)

	NH	Hα	Hβ2	Hβ3	Hγ2	Hγ3	Hδ2	Hδ3	Hε	Hε	Hζ
Met1		4.12	2.16		2.63						
Ala2	8.72	4.20	1.41 (Me)								
Ser3	8.27	4.40	3.92; 3.84								
His4	8.40	4.62	3.19				7.13 (C4H); 8.14 (C2H)				
Lys5	8.14	4.32	1.76								
Leu6	8.14	4.35	1.73		1.66		0.96 (Me)				
Leu7	7.93	4.34	1.72		1.66		0.92 (Me)				
Val8	7.68	4.24	2.22		0.96 (Me)						
Thr9	7.81	4.60	4.34		1.26 (Me)						
Pro10		4.42	2.07		2.35		3.83; 3.77				
Leu11	7.76	4.12	1.68		1.68		0.91 (Me)				
Lys12	7.95	3.94	1.90		1.70						
Ala13	7.73	4.35	1.42 (Me)								
Leu14	7.84	4.32	1.90		1.49		0.88 (Me)				
Leu15		4.19									

Lys16	7.74	4.33	1.92	1.75			
Pro17		4.43	2.10; 1.99	2.33		3.85; 3.64	
Leu18	7.94	4.34	1.80	1.63		0.94 (Me)	
Ser19	8.06	4.47	3.92				
Ile20	7.79	4.17	1.70; 0.89 (Me)		1.18	0.89 (Me)	
Pro21		4.40				3.64	
Tyr22	7.68	4.47	3.03			7.17	6.87

Table S7. Chemical shifts (δ, ppm from TSP) of NTT-AGT T9E in 30 % TFE (pH 7.2, 10 °C)

	NH	Hα	Hβ2	Hβ3	Hγ2	Hγ3	Hδ2	Hδ3	Hε	Hε	Hζ
Met1		4.18	2.18		2.66						
Ala2	8.82	4.42	1.47 (Me)								
Ser3	8.43	4.47	3.90								
His4	8.56		3.33; 3.23				7.30 (C4H); 8.57 (C2H)				
Lys5											
Leu6											
Leu7											
Val8											
Glu9[a]	8.45	4.42	1.93		2.32						
Pro10											
Pro11											
Lys12											
Ala13		4.22	1.43 (Me)								
Leu14											
Leu15											

Lys16
Pro17
Leu18
Ser19
Ile20
Pro21
Tyr22

[a] The signal in the TOCSY spectrum was very broad.

Table S8. Chemical shifts (δ, ppm from TSP) of NTT-AGT T9E-P11L in 30 % TFE (pH 7.2, 10 °C)

	NH	Hα	Hβ2	Hβ3	Hγ2	Hγ3	Hδ2	Hδ3	Hε	Hε	Hζ
Met1[a]		4.18	2.18		2.66						
Ala2	8.76	4.42	1.42 (Me)								
Ser3	8.31	4.43	3.91; 3.86								
His4	8.59	4.70	3.32; 3.22				7.28 (C4H); 8.54 (C2H)				
Lys5	8.30	4.26	1.82		1.46						
Leu6	8.12	4.36	1.63		1.63		0.90 (Me)				
Leu7	7.98	4.41	1.68		1.68		0.93 (Me)				
Val8	7.62	4.12	2.15		0.96 (Me)						
Glu9	8.37	4.41	2.12		2.38						
Pro10		4.42	2.08		2.33		3.63	3.81			
Leu11	7.73	4.17	1.87		1.87		0.92 (Me)				
Lys12	7.90	3.94	1.88								
Ala13	7.81	4.22	1.51 (Me)								
Leu14[a]	7.78	4.22	1.78		1.78		0.87 (Me)				
Leu15[a]	7.78	4.22	1.78		1.78		0.87 (Me)				

Lys16	7.81	4.22	1.94	1.35		
Pro17		4.34	2.09	2.35	3.95 3.68	
Leu18	7.88	4.36	1.83	1.65	0.92 (Me)	
Ser19	8.06	4.45	3.92			
Ile20	7.75	4.17	1.80; 0.91 (Me)	1.50 1.19	0.91 (Me)	
Pro21		4.44	1.96	2.24	3.59	
Tyr22[b]	7.67	4.51	3.09		7.17	6.89
	7.69	4.51	3.09		7.15	6.87

[a]Tentatively assigned.
[b]It shows two peaks.

Table S9. Thermodynamic parameters for TFE induced folding determined from a global two-state analysis. Errors are those from fittings.

NTT peptide	m (kcal·mol^{-1}·M^{-1})	C_m (M)	ΔG (kcal·mol^{-1})
**WT**	0.90±0.06	2.63±0.04	2.37±0.07
**P11L**	0.98±0.05	2.26±0.03	2.21±0.06
**T9E**	0.92±0.07	2.44±0.03	2.43±0.04
**T9E-P11L**	1.34±0.09	2.26±0.04	3.01±0.10
